# Heterologous DNA-prime/protein-boost immunization with a monomeric SARS-CoV-2 spike antigen redundantizes the trimeric receptor-binding domain structure to induce neutralizing antibodies in old mice

**DOI:** 10.3389/fimmu.2023.1231274

**Published:** 2023-09-11

**Authors:** Dominik Pflumm, Alina Seidel, Fabrice Klein, Rüdiger Groß, Lea Krutzke, Stefan Kochanek, Joris Kroschel, Jan Münch, Katja Stifter, Reinhold Schirmbeck

**Affiliations:** ^1^ Department of Internal Medicine I, University Hospital of Ulm, Ulm, Germany; ^2^ Institute of Molecular Virology, Ulm University Medical Center, Ulm, Germany; ^3^ Department of Gene Therapy, University Hospital of Ulm, Ulm, Germany; ^4^ Institute of Clinical Chemistry, Ulm University Medical Center, Ulm, Germany

**Keywords:** SARS-CoV-2, spike antigen, antigen conformation, vaccination regimens, antibody response, old mice

## Abstract

A multitude of alterations in the old immune system impair its functional integrity. Closely related, older individuals show, for example, a reduced responsiveness to severe acute respiratory syndrome coronavirus-2 (SARS-CoV-2) vaccines. However, systematic strategies to specifically improve the efficacy of vaccines in the old are missing or limited to simple approaches like increasing the antigen concentration or injection frequencies. We here asked whether the intrinsic, trimeric structure of the SARS-CoV-2 spike (S) antigen and/or a DNA- or protein-based antigen delivery platform affects priming of functional antibody responses particularly in old mice. The used S-antigens were primarily defined by the presence/absence of the membrane-anchoring TM domain and the closely interlinked formation/non-formation of a trimeric structure of the receptor binding domain (S-RBD). Among others, we generated vectors expressing prefusion-stabilized, cell-associated (TM^+^) trimeric “S2-P” or secreted (TM^−^) monomeric “S6-P_ΔTM_” antigens. These proteins were produced from vector-transfected HEK-293T cells under mild conditions by Strep-tag purification, revealing that cell-associated but not secreted S proteins tightly bound Hsp73 and Grp78 chaperones. We showed that both, TM-deficient S6-P_ΔTM_ and full-length S2-P antigens elicited very similar S-RBD-specific antibody titers and pseudovirus neutralization activities in young (2–3 months) mice through homologous DNA-prime/DNA-boost or protein-prime/protein-boost vaccination. The trimeric S2-P antigen induced high S-RBD-specific antibody responses in old (23-24 months) mice through DNA-prime/DNA-boost vaccination. Unexpectedly, the monomeric S6-P_ΔTM_ antigen induced very low S-RBD-specific antibody titers in old mice through homologous DNA-prime/DNA-boost or protein-prime/protein-boost vaccination. However, old mice efficiently elicited an S-RBD-specific antibody response after heterologous DNA-prime/protein-boost immunization with the S6-P_ΔTM_ antigen, and antibody titers even reached similar levels and neutralizing activities as in young mice and also cross-reacted with different S-variants of concern. The old immune system thus distinguished between trimeric and monomeric S protein conformations: it remained antigen responsive to the trimeric S2-P antigen, and a simple change in the vaccine delivery regimen was sufficient to unleash its reactivity to the monomeric S6-P_ΔTM_ antigen. This clearly shows that both the antigen structure and the delivery platform are crucial to efficiently prime humoral immune responses in old mice and might be relevant for designing “age-adapted” vaccine strategies.

## 1 Introduction

Aging-associated remodelling of the immune system largely goes along with an increased frequency and severity of infectious diseases and reduced humoral and cellular immune responses to vaccines, particularly when the old immune system is exposed to new antigens/pathogens (1, 2). Many aspects of impaired functionalities in different arms of the old immune system have been described and often were highly interlinked, like a disturbed CD4 T-cell signaling to B cells (3) and molecular defects in B cells that impair the presentation of foreign antigens and/or the priming of antibody responses (4–7). As a consequence, old individuals showed an increased risk of severe disease and a higher mortality upon severe acute respiratory syndrome coronavirus-2 (SARS-CoV-2) infection and a decreased responsiveness to vaccination (8–15). This decline in the responsiveness to new virus infections and/or vaccines was not limited to SARS-CoV-2 infection, since very different vaccine-induced antibody responses in the old, e.g., against influenza vaccines, are weaker and decline faster (1, 16, 17). Although SARS-CoV-2 infects people of all ages and genders, research showed that individuals with pre-existing comorbidities like hypertension, cardiovascular disease, obesity, diabetes, or cancer were more susceptible to COVID-19 infection (18, 19). Therefore, general vaccination strategies need to be developed to improve the efficacy of vaccines in the vulnerable elderly population.

Similar to other coronaviruses, the spike (S) glycoprotein of SARS-CoV-2 contains an NH_2_-terminal signal peptide sequence for targeting the endoplasmic reticulum (ER) and a COOH-terminal transmembrane domain (TM) for anchorage into membranes during protein biosynthesis. The membrane-spanning domain prevents it from being released into the lumen of the ER and being secreted. The nascent TM^+^ S protein thus is stably bound to the ER-membrane, where it assembles into trimeric structures and becomes co-translationally modified (e.g., glycosylated) during transport to the cell surface (20, 21). In particular, the TM domain is crucial for the formation of the trimeric receptor binding domain (S-RBD) structure (22, 23). The trimeric conformation of the S-RBD was essential for the binding of the virus to the angiotensin-converting enzyme 2 (ACE2) receptor and its uptake into host cells (8, 21, 24, 25). Therefore, the S protein and particularly the S-RBD were apparently the primary target for neutralizing antibodies (24–27) and thus an attractive vaccine antigen. However, the S protein is metastable, membrane anchored, and difficult to produce recombinantly in high amounts in different expression systems (28). In contrast, it was well established that genetically engineered TM-deficient S proteins of coronaviruses and SARS-CoV-2 were secreted into the cell culture supernatant, expressed at higher levels than full-length S proteins, but these monomeric proteins failed to assemble into trimeric structures unless modified with an artificial trimerization domain (24, 28–35). It is not clear to what extent conformational trimeric S-structures induce S- and/or S-RBD-specific neutralizing antibody responses, since neutralizing antibodies could also be primed by monomeric S-RBD- or S1-subunit antigens (32, 36–39). The S-specific antibody response thus might also be directed against conformation-independent domains within the S-RBD or against domains that do not cover the S-RBD at position S_319–541_ (24).

We primarily were interested to investigate whether, and to what extent, cell-associated transmembrane-bound trimeric (TM^+^) versus secreted transmembrane-deficient monomeric (TM^-^) S proteins elicit S-RBD-specific antibody responses in young and old mice through DNA- and protein-based immunization. We generated DNA vectors expressing TM^+^ and TM^−^ S proteins that were used for both production of the respective recombinant S proteins in transfected HEK-293T cells and DNA-based vaccination studies. We focused on the quantification of S-RBD-specific antibody titers by a commercial Elecsys Anti-SARS-CoV-2 S immunoassay and the determination of their neutralization capacity using an established SARS-CoV-2 pseudovirus platform (40, 41). We could show that both monomeric and trimeric S-antigens induced neutralizing antibodies in young (2–3 months) mice. High titers of neutralizing antibodies were also primed in old (23–24 months) mice by trimeric, but very inefficiently by monomeric S-antigens. Most interestingly, the efficacy of monomeric S-antigens to prime neutralizing antibodies in old mice was reconstituted by a heterologous DNA-prime/protein-boost vaccination regimen.

## 2 Materials and methods

### 2.1 Mice

Young (2–3 months) male and female C57BL/6J mice were purchased from Janvier (Le Genest-Saint-Isle, France). Furthermore, middle-aged (16 months) and old (23–24 months) male and female C57BL/6J mice (initially obtained from Janvier) were obtained from our in-house breeding colonies (SFB 1506 “Aging at interfaces”; project Z02) at the Tierforschungszentrum of Ulm University. Mice were routinely housed in our animal facility under standardized pathogen-free (SPF) conditions. Aging mice were routinely checked for overall appearance, and only healthy aged mice were used in the described experiments.

### 2.2 Construction of expression plasmids

The original SARS-CoV-2 spike sequence (S), encoding aa1-1273 of the Wuhan-Hu-1 strain (NCBI reference sequence: NC_045512.2), was codon optimized and synthesized by GeneArt (Thermo Fisher Scientific) and cloned into the pCI expression vector (cat. no. E1731, Promega) using NheI/NotI sites. This sequence was used to generate the other S-constructs described in this study by site-directed mutagenesis or Gibson Assembly. The respective primers for each mutagenesis PCR were ordered at Biomers.net GmbH (Ulm, Germany). The PCRs were conducted using 12.5 μl of the Phusion High-Fidelity PCR Master Mix (New England Biolabs, USA), 12.5 ng of the respective template, and 0.5 μM of the respective forward and reverse primer. PCR protocols, especially the annealing temperatures and the extension time, were adjusted to the respective primer pairs and constructs. The general PCR protocol was as follows: initial denaturation (98°C, 30 s), 20× denaturation (98°C, 10 s), annealing (62°C–72°C, 30 s), extension (72°C, 180 s–420 s) and final extension (72°C, 600 s). All antigen constructs used for protein- and DNA-based vaccination studies were fused with a COOH-terminal Strep-tag sequence (SWSHPQFEKGGGSGGGSGGGSWSHPQFEK). Furthermore, we designed a vector encoding a S6-P_ΔTM/EPEA_ detection antigen for S-specific ELISA (see below) by adding a COOH-terminal linker sequence and a glutamic acid–proline–glutamic acid–alanine tag sequence (GYQDY-EPEA). Plasmids were produced in transformed *E. coli* DH5α (Thermo Fisher Scientific, USA) and purified using the endotoxin-free Plasmid Mega Kit (#12183; Qiagen). All plasmids were sequenced before use by Eurofins Genomics (Germany) to ensure the correct sequence of the respective construct.

### 2.3 Recombinant protein production and purification

S proteins were expressed in HEK293T cells (ATCC CRL-3216) transiently transfected for 48 h with the indicated plasmid DNAs using the calcium phosphate method (42). Usually, approximately 5×10^8^ cells were used for large-scale production of the indicated cell-associated and secreted antigens. Cells were cultured in Dulbecco´s Modified Eagle Medium (DMEM) high-glucose medium supplemented with 10% heat-inactivated Fetal calf serum (FCS), 1% Penicillin/Streptomycin, 1% L-glutamine, and 0.1% β-mercaptoethanol at 37°C/5% CO_2_. At 24 h post-transfection, the medium was changed, and at 48 h post-transfection, cell culture supernatants were collected, cleared by centrifugation (450×*g*, 5 *min*), and subsequently passed through a 0.45-µm filter. Notably, the expression conditions for the production of the secreted S6-P_ΔTM_ were further optimized. At 24 h post-transfection, the culture medium was additionally supplemented with 3.75 mM valproic acid and 3 g/L D-(+)-glucose (43), and cells were cultured for additional 5 days before harvesting the supernatant.

For purification of cell-associated/membrane-anchored S-antigens, cells were lysed for 30 min at 4°C with 5 ml of lysis buffer [100 mM Tris–HCl, 150 mM NaCl, 0.5% NP40 detergent, and Protease Inhibitor Cocktail Tablets (cat. no. 11836145001, Roche Applied Science; Penzberg, Germany) pH 8.0] and cleared by centrifugation (8,000×*g*, 30 *min*). Recombinant antigens in cell lysates and supernatants were purified with Strep-Tactin (IBA Lifesciences, Germany) as described previously (42). Briefly, cell extracts or supernatants were passed over StrepTactin Sepharose (#2-1201-025, IBA, Göttingen, Germany) packed polypropylene columns (cat. no. 29922, Pierce, Rockford, USA). Sepharose-bound antigens were purified with five column volumes of wash buffer (100 mM Tris–HCl, 150 mM NaCl, 1mM EDTA; pH 8.0) and eluted in eight 500-µl fractions in elution buffer (20 mM Tris–HCl, 30 mM NaCl, 0.2mM EDTA; pH 8.0) supplemented with 2.5 mM desthiobiotin (cat. no. 2-1000-002, IBA, Göttingen, Germany). S-protein-containing fractions were determined by sodium dodecyl sulfate polyacrylamide gel electrophoresis (SDS-PAGE) and Coomassie Brillant Blue staining of the gels (see also **Supplementary Figure S1**). S-protein-containing samples were pooled and concentrated in Vivaspin 500 columns (30,000 or 100,000 MW cutoff) (cat. no. VS0141; Sartorius; UK) to a volume of approximately 100–200μl. Furthermore, an S6-P_ΔTM/EPEA_ detection antigen used for S-specific ELISA (see below), modified with a COOH-terminal linker sequence and a glutamic acid–proline–glutamic acid–alanine tag sequence (GYQDY-EPEA), was purified from cell culture supernatants using CaptureSelect™ C-tagXL Affinity Matrix (Thermo Fisher Scientific, USA) according to the manufacturer’s instructions (https://assets.thermofisher.com/TFS-Assets/LSG/manuals/MAN0017302_CapSelCtagXLAffMatrix_PI.pdf). We also purchased a recombinant, carrier-free trimeric S2-P_ΔTM/GCN4-IZ_ SARS-CoV-2 S protein (accession no. YP_009724390.1) (cat. no. 10561-CV; R&D Systems). This S protein was produced in HEK293 cells, contains the ectodomain up to aa1211, a silenced furin cleavage site (S_R682S,R685S_), a prefusion stabilization motive (S_K986P,V987P_), a COOH-terminal GCN4-IZ trimerization domain, and a 6x-His-tag.

### 2.4 SDS-PAGE, Western blot analyses, and size exclusion chromatography

Samples of recombinant S proteins were either mixed with a fourfold concentrated SDS loading buffer (250 mM Tris–HCl (pH 6.8), 4% SDS, 40% glycerol, 0.02% bromphenol blue) either with (reducing) or without (non-reducing) 200 mM β-mercaptoethanol. Samples were mixed with the indicated loading buffer at a ratio of 3:1 (v/v). Only the samples analyzed under reducing conditions were additionally boiled (95°C for 5 min). Afterwards, the samples were loaded onto a 10% Bis–Tris gel using the Mini-PROTEAN electrophoresis chamber (Bio-Rad, USA). Gels were stained with Coomassie Brilliant Blue (44.8% methanol, 9.2% acetic acid, and 2 mM Coomassie R-250) for 30 min and destained in a mixture of 10% methanol and 10% isopropanol for 60–120 min at room temperature.

For Western blotting, the gels were blotted onto a 0.2µm nitrocellulose membrane in transfer buffer (25 mM tris, 200 mM glycine, and 15% methanol) at 60 V for 60 min using the Mini Trans-Blot Cell system (Bio-Rad, USA). Thereafter, the membranes were incubated with blocking buffer for 60 min at room temperature (1% milk powder in PBS), washed twice with washing buffer (15 mM Tris–HCl, 150 mM NaCl, 5 mM sodium azide, 1 mM EDTA, and 0.1% Tween-20; pH 7.8) and incubated with primary antibodies for 60 min at room temperature: anti-Strep-tag (1:33.333; StrepMAB-Classic-HRP, IBA Lifesciences, Germany), anti-Grp78/BiP (1:5,000, Proteintech, USA), anti-Hsc70/Hsp73 (1:1,000, Enzo Life Sciences, USA), or mouse anti-S sera (1:500) derived from mice vaccinated twice with pCI/S2-P_ΔTM_ DNA, and detected with the respective secondary antibodies, namely, anti-mouse IgG-HRP (1:2,500, Amersham, UK) or anti-rabbit IgG-HRP (1:2,000, Amersham, UK). Blots were developed for 15–30 s using the Immobilon Western Chemiluminescent HRP Substrate (Merck Millipore, USA) and documented using the Fusion FX (Vilber Lourmat, France).

Size exclusion chromatography (SEC) experiments were performed with Superose 6 Increase 10/300 GL column (Cytiva 29-0915-96). All runs were performed with PBS buffer at 0.5 ml/min after injecting 250-µl spike constructs (0.5 mg/ml). The column was calibrated using a mixture of dextran blue (MW ≥ 2MDa) and globular proteins of known molecular weights of 670 kDa, 158 kDa, 44 kDa, 17 kDa, and 1.35 kDa (cat no. 151-1901, Biorad gel-filtration calibration kit). The calibration was used to estimate the apparent molecular weight of the different S-species analyzed by SEC (Kav method).

### 2.5 Immunization of mice

Mice were immunized i.m. into both tibialis anterior muscles at day 0 (prime) and day 22 (boost) with 100 µg DNA diluted in 50 µl PBS and/or 10 µg of protein mixed with 10 µg Quil-A adjuvant in 50 µl PBS (provided by Dr. Eric Lindblad, Brenntag Biosector, Frederikssund, Denmark). Blood samples were collected at day 36 (day 14 post-boost).

### 2.6 Determination of S- and S-RBD-specific antibody titers

S-RBD-specific antibody titers were routinely determined using the commercially available Elecsys anti-SARS-CoV-2 S immunoassay (Roche, Switzerland) applied on a cobas pro e 801 module (Roche, Switzerland) in the Institute of Clinical Chemistry, Ulm University Medical Center. Briefly, serum samples were diluted 1:10 in PBS to a final volume of 150 µl and analyzed according to the manufacturer’s instructions. The raw values were corrected with the dilution factor and depicted as S-RBD-specific IgG/IgM antibody titers (U/ml). The dotted lines in the respective graphs showing S-RBD-specific IgG/IgM antibody titers in (U/ml) represent the limit of quantification (0.8 U/ml) as described by the manufacturer of the test. The assay was validated by the “Clinical Chemistry” of the Ulm University Medical Center. Confirmatory, all negative control sera derived from unimmunized young and old control mice resulted in a value below 0.8 U/ml.

Where indicated, S-antigen-specific IgG antibody titers were measured with the secreted recombinant S6-P_ΔTM/EPEA_ detection antigen by endpoint ELISA. This protein was used to exclude any cross-reactivities against the strep-tag. Briefly, Nunc MaxiSorp™ 96-well plates (Thermo Fisher Scientific, USA) were coated with 0.1 µg of S6-P_ΔTM/EPEA_/well diluted in 0.1 M sodium carbonate buffer (pH 9.5) and incubated at 4°C overnight, washed (0.05% Tween-20 in PBS), and incubated with blocking buffer (3% BSA in PBS). Serial dilutions (1:2) of serum samples derived from unimmunized young or old control and vaccinated mice (starting at 1:30 to a dilution of maximal 409,600) were added for 2 h at room temperature, washed, incubated for 1 h at 37°C with anti-mouse IgG-HRP (BD Biosciences, USA), developed with 0.4 mg/ml OPD (Merck) and 0.012% hydrogen peroxide dissolved in 0.05 M citrate-phosphate buffer (pH 5.0). The samples were then analyzed at 492 nm using a 96-well plate reader (MWG Biotech, Germany). The S-specific endpoint titers were defined as the highest serum dilution that resulted in an absorbance value three times greater than that of control sera from unimmunized young or old mice.

### 2.7 Production of pseudotyped particles

The production of pseudotyped particles has been previously described (40, 41). In brief, HEK293T cells were transfected with plasmids encoding SARS-CoV-2 S variants B.1, B.1.1.7 (Alpha), B.1.351 (Beta), B.1.617.2 (Delta), or B.1.1.529.1 (Omicron BA.1) (44–46) by Transit LT-1 (Mirus, USA). After 24 h, cells were inoculated with a replication-deficient vesicular stomatitis virus (VSV) vector in which the genetic information for its native glycoprotein (VSV-G) is replaced by genes encoding enhanced green fluorescent protein and firefly luciferase (kindly provided by Gert Zimmer, Institute of Virology and Immunology, Mittelhäusern, Switzerland) and incubated at 37°C for 2 h. Then, the inoculum was removed, cells were washed with PBS, and fresh medium containing anti-VSV-G antibody (from I1-hybridoma cells; ATCC no. CRL-2700) was added to block residual VSV-G carrying particles. After 16–18 h, supernatants were collected and cleared by centrifugation. Samples were aliquoted and stored at −80°C.

### 2.8 Pseudovirus neutralization assay

The antibody-mediated SARS-CoV-2 spike-specific pseudovirus neutralization assay was performed as described previously (40, 41). Briefly, Vero E6 cells (6,000 cells/well) were seeded in 96-well plates 1 day prior in DMEM supplemented with 2.5% FCS, 100 U/ml penicillin, 100 μg/ml streptomycin, 2 mM L-glutamine, 1 mM sodium pyruvate, and 1× non-essential amino acids. Heat-inactivated (56°C, 30 min) serum samples were serially titrated in PBS (fourfold titration series with seven steps plus buffer only control) and mixed with VSV∗ΔG-FLuc pseudovirus stocks (1:1, v/v) in a total volume of 48 µl. Serum-pseudovirus mixes were incubated for 30 min at 37°C before being added to the Vero E6 target cells in duplicates (final on-cell dilution of sera: 100-, 400-, 1,600-, 6,400, 25,600-, 102,400-, 409,600-fold). After 16–18 h, the transduction efficiency was analyzed. Cells were lysed with Cell Culture Lysis Reagent (cat. no. E1531; Promega) at room temperature. Lysates were then transferred into white 96-well plates (cat. no. 136101; Thermo Fisher), and luciferase activity was measured using a Luciferase Assay System (cat. no. E1501; Promega) and a plate luminometer (Orion II Microplate Luminometer, Berthold). For quantitative analysis of raw values, the background signal of untreated control cells (in relative light units per second; RLU/s) was subtracted, and values were normalized to pseudovirus-infected control cells in the absence of serum. Results are given as serum dilution factors resulting in 50% pseudovirus neutralization (50% pseudovirus neutralizing titer; PVNT50) on cells, calculated by nonlinear regression ([inhibitor] vs. normalized response–variable slope) in GraphPad Prism Version 9.1.1. As the tested serum dilutions range from 100- to 409,600-fold, we could detect PVNT50 titers within this range, defining the lower and upper cutoff values as 100 and 409,600, respectively. The dotted lines in the respective graphs showing PVNT50 values represent the limit of quantification (100).

### 2.9 Statistical analysis

The GraphPad Prism 8.4.3 software (GraphPad, San Diego, CA, USA) was used for statistical analyses and creation of graphs. Statistically significant differences between two indicated groups were determined using Student’s unpaired *t*-test. Where indicated, we also used Kruskal–Wallis followed by Dunn’s multiple comparisons test. p-values smaller than 0.05 were considered as statistically significant and indicated with asterisks in the graphs (p<0.05*, p<0.01**, and p<0.001***). Non-significant (ns) differences between individual groups were not marked in the graphs and described in the respective figure legends. Data are depicted as geometric mean ± geometric SD, and group sizes are stated in the figure legends.

## 3 Results

### 3.1 Characterization of cell-associated, transmembrane-anchored (TM^+^) versus secreted transmembrane-deficient (TM^−^) S antigens

**Figure 1:**
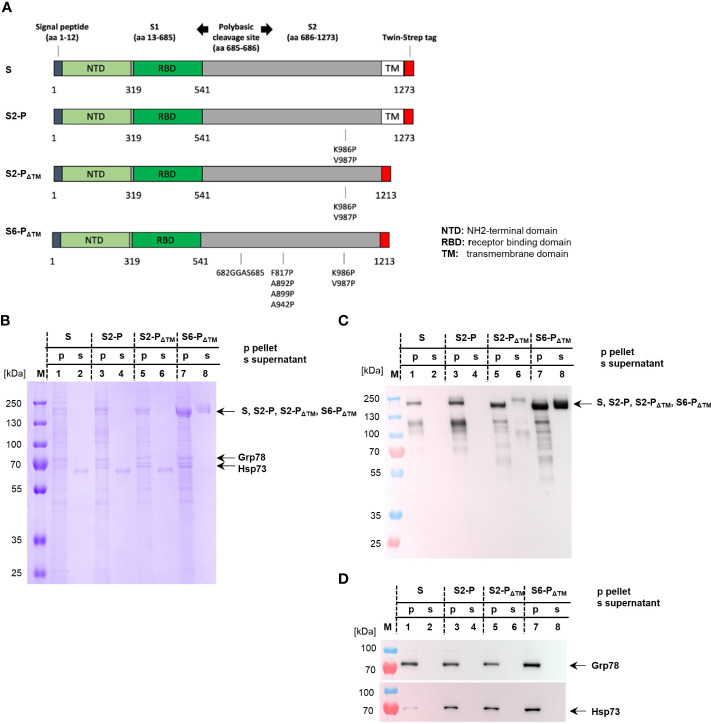
**(A)** Map of the different SARS-CoV-2 S proteins cloned into the pCI expression vector. The positions of S1 and S2, the signal peptide, the NH_2_-terminal domain (NTD), the receptor-binding domain (RBD), the COOH-terminal transmembrane (TM) domain, and the position of the Strep-tag sequence within the S protein (S) are shown. The positions of the amino acid exchanges at S_K986P_ and S_V987P_ generating the prefusion-stabilized S2-P (S2-P) and its TM-deficient S2-P_ΔTM_ variant (S2-P_ΔTM_) are shown. Furthermore, the amino acid substitutions S_K986P,V987P,F817P,A892P,A899P,A942P_ and S_R682G,R683G,R685S_ (depicting the furin cleavage site) generating the TM-deficient S6-P_ΔTM_ are indicated. **(B–D)** HEK-293T cells were transiently transfected with pCI/S (lanes 1 and 2), pCI/S2-P (lanes 3 and 4), pCI/S2-P_ΔTM_ (lanes 5 and 6), and pCI/S6-P_ΔTM_ (lanes 7 and 8), followed by Strep-tag purification of the respective proteins from the cell lysate/pellet (p; lanes 1, 3, 5, and 7) or the cell culture supernatant (s; lanes 2, 4, 6, and 8). The purified S proteins were obtained from either 10×10^8^ (pCI/S-2P, pCI/S), 7×10^8^ (pCI/S2-P_ΔTM_), or 5×10^8^ (S2-P_ΔTM_) HEK-293T cells. The S proteins were purified and processed for SDS-PAGE under reducing conditions, followed by Coomassie Brilliant Blue staining of the gels **(B)** or specific Western blot analyses using sera from pCI/S2-P_ΔTM_-immune mice **(C)** or Hsp73- and Grp78-specific antibodies **(D)**. The positions of the respective monomeric S protein bands (S, S2-P, S2-P_ΔTM_; S6-P_ΔTM_) of Hsp73 and Grp78 are indicated.

To investigate the antigenicity of cell-associated trimeric and secreted monomeric S proteins through DNA- and protein-based immunization, we generated mammalian expression vectors that were used for both the production of recombinant S proteins in transiently transfected HEK-293T cells and DNA-based vaccination studies. We generated vectors encoding the TM-anchored full-length S protein from the original Wuhan-Hu-1 strain (pCI/S) and a mutant S2-P with two proline substitutions at positions S_K986P_,_V987P_ that convey a superior conformation and antigenicity by stabilization of the prefusion conformation (pCI/S2-P) (47, 48) (**Figure 1A**). Considering that TM-deficient S proteins were efficiently secreted into the cell culture supernatant, but did not form higher molecular trimeric structures unless modified with a specific trimerization domain (28, 49, 50), we designed vector DNAs encoding a prefusion stabilized (S_K986P_,_V987P_) TM-deficient and secreted S2-P_ΔTM_ (pCI/S2-P_ΔTM_) protein and a derivative thereof with four additional proline substitutions (S_K986P,V987P,F817P,A892P,A899P,A942P_) (29) and a silenced furin cleavage site (S_R682G,R683G,R685S_) ensuring structural stability (pCI/S6-P_ΔTM_) (**Figure 1A**). Furthermore, to enable standardized affinity purification of the respective S proteins and detection of intrinsic structures or possible interactions with cellular proteins, we cloned a Strep-tag sequence at the COOH-termini of the S-sequences (**Figure 1A**). The Strep-tag purification system was used because we had already confirmed its capability to produce recombinant antigens, like RNA-binding proteins or protein particles, under very mild conditions with high purity (42). We thus expected to express and produce different monomeric and trimeric S proteins and to reveal if *de novo* expressed TM^+^ and TM^-^ S proteins interact with cellular proteins, an issue that could mimic antigen expression in antigen-presenting cells upon DNA-based vaccination and therefore impact the efficacy of the respective vaccines (51–53).

For large-scale production of recombinant proteins, usually 5–10×10^8^ HEK-293T cells were transiently transfected with the different vector DNAs, followed by Strep-Tactin-specific purification of the respective S proteins from cell lysates or cell culture supernatants. A representative SDS-PAGE analysis of the purification of the secreted S6-P_ΔTM_ is shown in **Supplementary Figure S1**. Samples were subjected to SDS-PAGE under reducing conditions followed by Coomassie Brilliant Blue staining of the gels (**Figure 1B**) or Western blot analyses using S-protein-specific antisera from vaccinated mice (**Figure 1C**). These analyses showed prominent steady-state levels of protein bands of approximately 180 kDa present in the cell lysates of all four constructs, depicting the S, S2-P, and the slightly smaller TM-deficient S2-P_ΔTM_ and S6-P_ΔTM_ proteins (**Figures 1B, C**). Western blot analysis but not Coomassie Brillant Blue staining of the gel also revealed a protein band of approximately 120 kDa that could not yet be identified (**Figures 1B, C**). As expected, the S and S2-P antigens were exclusively found in the cell lysates, whereas the S2-P_ΔTM_ and S6-P_ΔTM_ antigens were also detected in the cell culture supernatant (**Figures 1B, C**). However, the S6-P_ΔTM_ showed a much better secretion efficacy into the culture supernatant than the S2-P_ΔTM_ (**Figures 1B, C**). Therefore, we only used the S6-P_ΔTM_ in our vaccination studies. After optimizing the antigen expression conditions in transfected HEK-293T cells, we could gain approximately 600 μg of affinity-purified S6-P_ΔTM_ from the supernatant of 5×10^8^ transfected cells. Notably, it was difficult to reproducibly produce the cell-associated S and S-2P proteins, and on average, we could gain approximately 15–30 µg S and 30 µg S-2P proteins from cellular extracts of 10×10^8^ HEK-293T cells. Interestingly, Coomassie Brilliant Blue staining of the gels also revealed a prominent co-purification of additional 70–80 kDa protein bands with cellular S, S2-P, S2-P_ΔTM_, and S6-P_ΔTM_, but not secreted S2-P_ΔTM_ and S6-P_ΔTM_ proteins. (**Figure 1B**). These bands were identified by commercial NH_2_-terminal protein sequencing (TopLab; Munich, Germany) or Western blot analyses as ER-associated Grp78 and constitutively expressed cytosolic Hsp73 (**Figures 1B, D**). The cell-associated, but not the secreted S proteins thus stably bound Hsp73 and Grp78 (**Figures 1B, D**).

**Figure 2:**
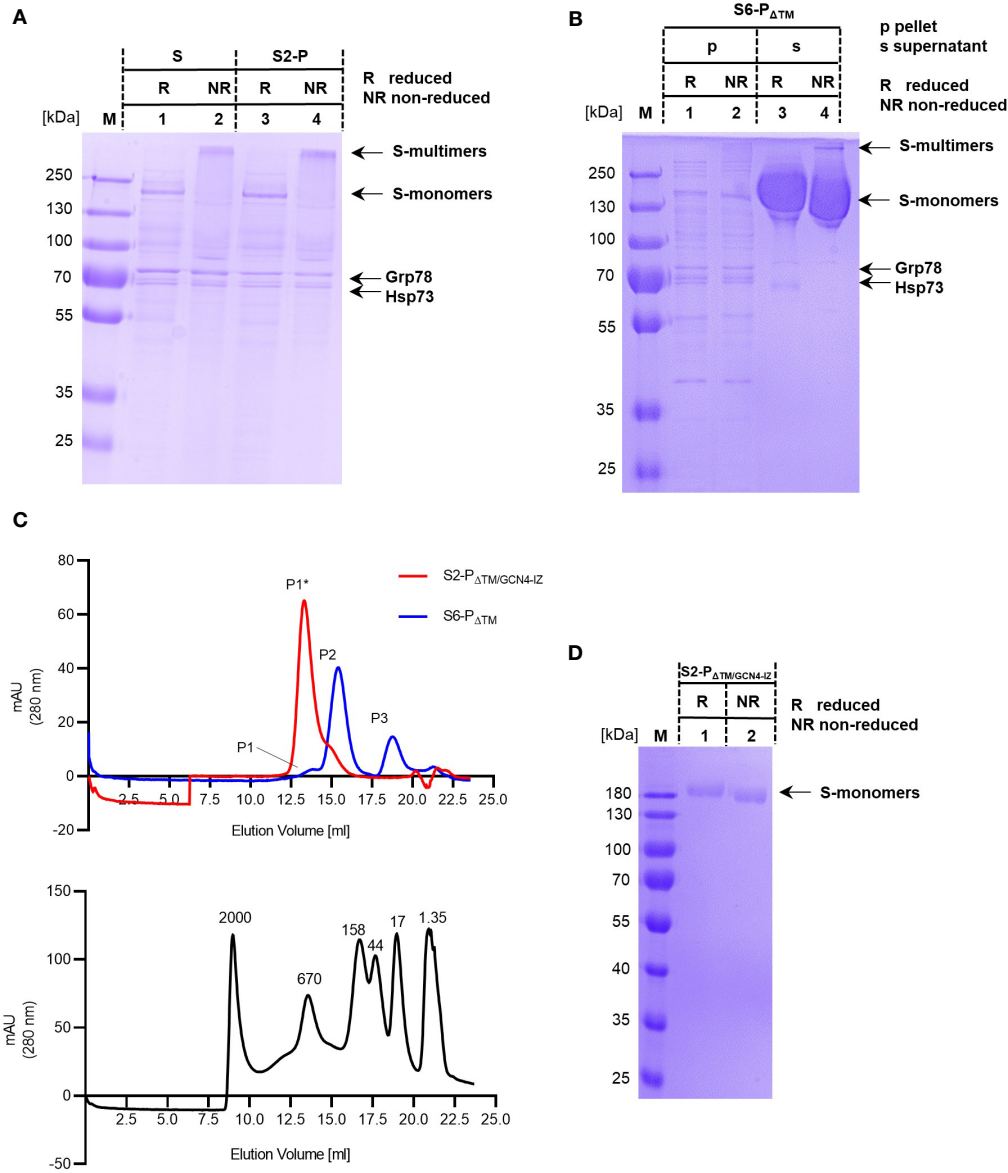
**(A, B)** Samples of purified S and S2-P **(A)** and of cell-associated (p) and secreted (s) samples of S6-P_ΔTM_ **(B)** were analyzed by SDS-PAGE under reducing (R) and non-reducing (NR) conditions. The positions of the respective monomeric (S-monomers) and high molecular (S-multimers) are shown. The S, S2-P, and S6-P_ΔTM_ protein bands and of Hsp73 and Grp78 are indicated. **(C)** Equal amounts (125 µg) of recombinant S6-P_ΔTM_ (blue line) or a trimeric S2-P_ΔTM/GCN4-IZ_ protein (red line) were analyzed by size exclusion chromatography (SEC) using a Superose 6 Increase 10/300 GL column (upper panel). The column was further calibrated with a mixture of dextran blue (MW ≥ 2MDa) and globular proteins of known molecular weights (670, 158, 44, 17, and 1.35 kDa) (lower panel). The main peaks, i.e., peak P1* corresponds to the approximately 670 kDa S2-P_ΔTM/GCN4-IZ_ trimers, and peak P2 corresponds to the approximately 370 kDa S6-P_ΔTM_ monomers. Furthermore, peak P1 corresponds to a high molecular protein of approximately 650 kDa and peak P3 to a small protein of approximately 17 kDa. **(D)** The trimeric recombinant S2-P_ΔTM/GCN4-IZ_ protein was analyzed by SDS-PAGE under reducing (R) and non-reducing (NR) conditions. The position of the respective protein bands (S-monomers) are indicated.

When non-reducing conditions were applied for SDS-PAGE, the 180-kDa full-length S and S2-P protein bands were no longer detectable and instead appeared as a high molecular band (**Figure 2A**, lanes 2 and 4), suggesting that these proteins maintained a high molecular multimeric structure. Furthermore, we observed in these non-reduced samples that anionic SDS-PAGE conditions were sufficient to strip off the stress proteins from the S-multimers appearing in the gels as separate bands (**Figure 2A**, lanes 2 and 4). In contrast, the prominent 180 kDa protein band of the secreted S6-P_ΔTM_ formed under both reducing and non-reducing conditions of SDS-PAGE (**Figure 2B**), indicating that the secreted S6-P_ΔTM_ was mainly composed of monomers (34, 49, 50). A very low amount of S6-P_ΔTM_ assembled into high molecular structures detectable in SDS-PAGE under non-reducing conditions (**Figure 2B**, lanes 3 and 4). Notably, an almost identical pattern of protein migration in SDS-PAGE has been shown for full-length, trimeric (TM^+^), and monomeric (TM^−^) coronavirus S proteins (34).

The affinity-purified secreted S6-P_ΔTM_ was further analyzed by size exclusion chromatography (SEC). As expected from SDS-PAGE analyses, we observed a SEC elution profile with one main protein peak of approximately 370 kDa as determined with globular standard proteins of known molecular weight (**Figure 2C**). Furthermore, minor protein peaks corresponding to a high molecular protein of approximately 650 kDa and a small protein of approximately 17 kDa were detectable (**Figure 2C**). To relate the individual protein peaks to S protein trimers, we used a commercially available, trimeric S2-P_ΔTM_ protein modified with a GCN4-IZ trimerization motif (S2-P_ΔTM/GCN4-IZ_). Analytical SEC showed a single peak of recombinant S2-P_ΔTM/GCN4-IZ_ corresponding to a S-trimer of approximately 670 kDa (**Figure 2C**). As expected, the trimeric S2-P_ΔTM/GCN4-IZ_ protein thus clearly separated from the smaller S6-P_ΔTM_ (**Figure 2C**), confirming that these proteins have two distinct conformations (49, 50). However, these findings also showed that it was difficult to determine the exact molecular weight of these two glycoproteins by SEC and simply assign it to the molecular weights expected from SDS-PAGE (i.e., considering an approximately 180 kDa molecular weight of a monomer). In our system, we determined higher molecular weights for both the trimeric S2-P_ΔTM/GCN4-IZ_ antigen and the monomeric S6-P_ΔTM_ antigen that differ from the calculated values, i.e., 670 kDa instead of 540 kDa for the trimer and 370 kDa instead of 180 kDa for the monomer. This was not unexpected, since their migration behavior during SEC could be affected among others by their glycosylation state possibly leading to an overestimation of the molecular weight up to 100% (54), their conformation, and from the molecular weight standard proteins (49, 50). Furthermore, the level of glycosylation might vary depending on the antigen itself, the cells used for production, and the level and/or time of expression.

An interesting observation was that, in contrast to full-length S- and S-2P proteins, the recombinant trimeric S2-P_ΔTM/GCN4-IZ_ protein was sensitive to anionic SDS-PAGE conditions and quantitatively disassembled into the typical monomeric 180 kDa protein band in both reducing and non-reducing SDS-PAGE (**Figure 2D**). This suggested that the natural TM domain, but not artificial trimerization motives, mediated a better stabilization of the high molecular S-trimers and their resistance to anionic SDS-PAGE conditions.

### 3.2 Priming of S-RBD-specific antibody responses in young mice by DNA- and protein-based immunization

**Figure 3:**
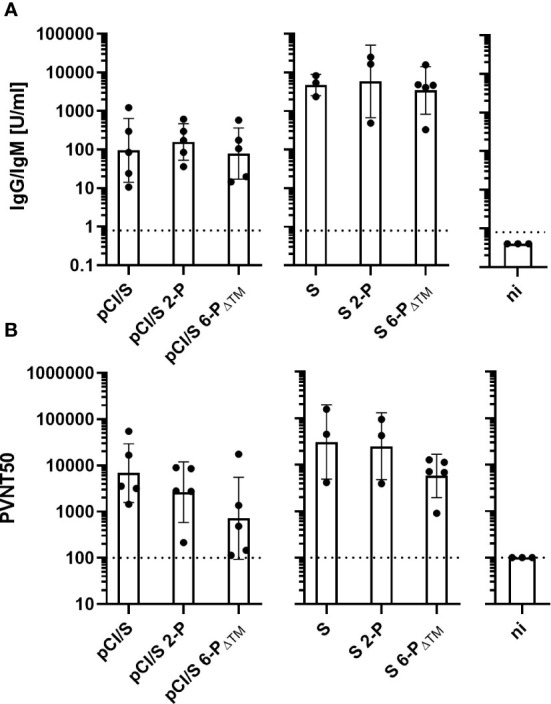
Young (2–3 months) C57BL/6J mice were immunized twice (at day 0 and 22) with 100 μg of plasmid DNA (pCI/S, pCI/S2-P, or pCI/S6-P_ΔTM_) (n=5) or 10 µg of recombinant protein/10 μg Quil-A, i.e., cellular S or S2-P, secreted S6-P_ΔTM_ (n=3–5). **(A)** S-RBD-specific serum antibody titers induced in DNA- and protein-immunized mice were determined with a commercial Elecsys Anti-SARS-CoV-2 S immunoassay at day 14 after the second vaccination. IgG/IgM antibody titers are shown in U/ml. As a control, sera from three non-immunized (ni) young mice were tested in the assay. Dotted lines represent the limit of quantification (0.8 U/ml). **(B)** The indicated serum samples were further tested in a vesicular stomatitis virus (VSV)-based SARS-CoV-2 S-carrying pseudovirus system to determine the neutralizing activity of elicited antibodies. The results are depicted as serum dilution factors that result in 50% pseudovirus neutralization (PVNT50). As a control, three sera from non-immunized (ni) young mice were tested in the neutralization assay. Dotted lines represent the detection limit of PVNT50 values (100). **(A, B)** The statistical significance of antibody titers **(A)** and PVNT50 values **(B)** induced by DNA and protein vaccines were determined using Kruskal–Wallis test followed by Dunn’s multiple comparisons test. p-values smaller than 0.05 were considered as statistically significant. All comparisons of antibody titers **(A)** and PVNT50 values were found to be not significant. Data are shown as geometric mean ± geometric SD.

To determine the antigenicity of cell-associated vs. secreted S proteins, we immunized young (2–3 months) C57BL/6J mice twice (at day 0 and 22), either with equal amounts of pCI/S, pCI/S-2P, or pCI/S6-P_ΔTM_ DNA and the recombinant, cell-associated S and S-2P- or secreted S6-P_ΔTM_ proteins in saponin-based Quil-A adjuvant (55) (**Figure 3**). S-RBD-specific antibody titers were measured with the commercial Elecsys Anti-SARS-CoV-2 S immunoassay at day 14 after the second vaccination (**Figure 3A**). Immunization of B6 mice with pCI/S, pCI/S2-P, or pCI/S6-P_ΔTM_ DNA induced very similar S-RBD-specific antibody titers (**Figure 3A**). This showed that neither the stabilization of the prefusion conformation in the pCI/S-2P vs. the pCI/S constructs (47, 48) nor the expected trimeric (pCI/S-2P and pCI/S) vs. monomeric (pCI/S6-P_ΔTM_) antigen structure had a striking effect on the priming of S-RBD-specific antibody responses in young mice through DNA-based immunization (**Figure 3A**). Furthermore, equal amounts of recombinant S and S2-P proteins isolated from cell lysates of transfected HEK-293T cells and secreted monomeric S6-P_ΔTM_ induced comparable S-RBD-specific antibody titers in young mice (**Figure 3A**). The differences in the antibody titers induced through the different DNA- and protein-based vaccines did not reach statistical significance, although recombinant proteins showed a pronounced trend to induce higher antibody titers than vector DNAs at the dosages used here (**Figure 3A**). Notably, the amount of recombinant S6-P_ΔTM_ protein and its formulation with adjuvant was crucial for the magnitude of antibody titers (**Supplementary Figure S2**).

We next used a vesicular stomatitis virus (VSV)-based SARS-CoV-2 pseudovirus carrying the S protein to determine the neutralizing activity of the elicited antibodies (40, 41). Irrespective of the antigen conformation, the S-specific antibodies developed in different DNA- (pCI/S, pCI/S2-P, pCI/S6-P_ΔTM_) or protein-immunized (S, S2-P, S6-P_ΔTM_) young mice efficiently suppressed S-mediated cell entry of pseudovirus particles (**Figure 3B**). This showed that both trimeric and monomeric S proteins elicited substantial pseudovirus-neutralizing antibodies in young mice, confirming previous reports using recombinant S1 or S-RBD subunit vaccines (28, 32, 36–39).

**Figure 4:**
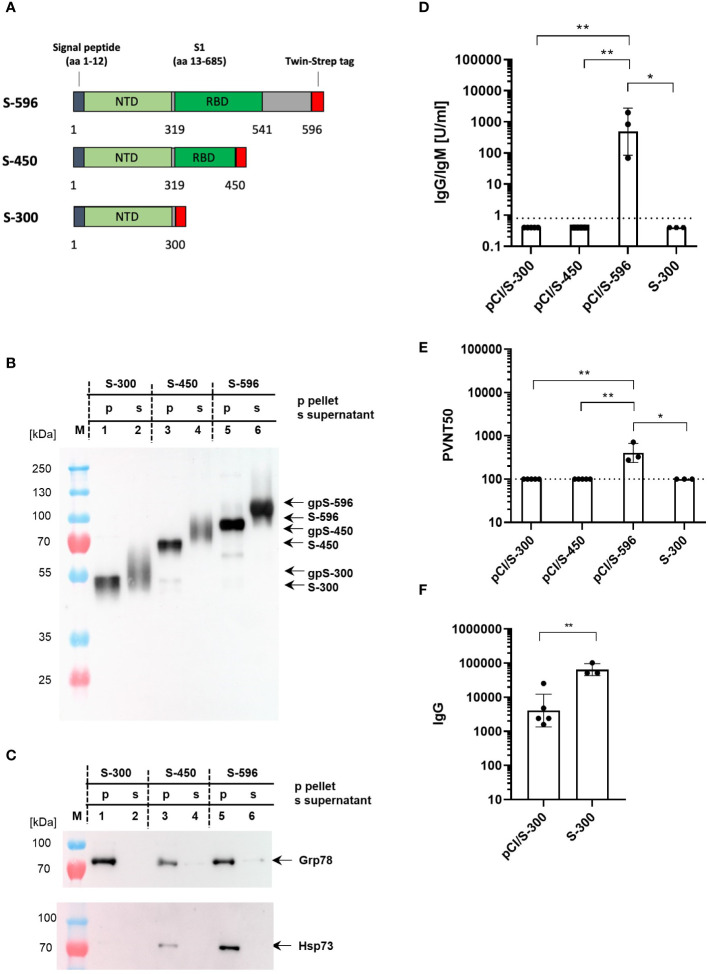
**(A)** Map of different S-300, S-450, and S-596 fragments, which were cloned into the pCI expression vector. The positions of the signal peptide, the NH_2_-terminal domain (NTD), the receptor-binding domain (RBD), and the Strep-tag sequence are shown. **(B, C)** 5×10^8^ HEK-293T cells were transiently transfected with pCI/S-300 (lanes 1 and 2), pCI/S-450 (lanes 3 and 4), and pCI/S-596 (lanes 5 and 6), followed by Strep-tag purification of the respective proteins from either the cell lysates (p; lanes 1, 3, and 5) or the cell culture supernatants (s; lanes 2, 4, and 6). A total of 300 ng S-300, 250 ng S-450, and 250 ng S-596 cell-associated (pellet) S proteins and 300 ng S-300, 300 ng S-450 and 250 ng S-596 secreted (supernatant) S proteins were subjected to SDS-PAGE followed by Western blot analyses using anti-Strep-tag **(B)** or anti-Hsp73- or anti-Grp78-specific antibodies **(C)**. The positions of cell-associated (S-300, S-450, S-596) and secreted, glycosylated (gpS-300, gpS-450, gpS-596) S antigen fragments, and of Hsp73 or Grp78 are indicated. **(D, E)** Young (2–3 months) C57BL/6J mice (n=3–5) were immunized twice (at day 0 and 22) with equal amounts of plasmid DNA (100 µg; pCI/S-300, pCI/S-450 or pCI/S-596) or 10 µg recombinant S-300/Quil-A protein, and S-RBD-specific serum antibody titers were determined with commercial Elecsys anti-SARS-CoV-2 S immunoassay at day 14 after the second vaccination. IgG/IgM antibody titers are shown in U/ml. Dotted lines represent the limit of quantification (0.8 U/ml) **(D)**. The indicated serum samples were further tested in a vesicular stomatitis virus (VSV)-based SARS-CoV-2 S-carrying pseudovirus to determine the neutralizing activity of elicited antibodies. The results are depicted as serum dilution factors that result in 50% pseudovirus neutralization (PVNT50). Dotted lines represent the detection limit of PVNT50 values (100) **(E)**. **(D, E)** Statistical significance between all groups were determined using Kruskal–Wallis test followed by Dunn’s multiple comparisons test. p-values smaller than 0.05 were considered as statistically significant and indicated with asterisks in the graphs (p<0.05*, p<0.01**). Data are shown as geometric mean ± geometric SD. **(F)** Serum samples derived from pCI/S-300 or S-300 immunized mice (described in **D, E**) were further analyzed for S-specific IgG antibody titers by an end-point ELISA. The S-specific endpoint titers were defined as the highest serum dilution that resulted in an absorbance value three times greater than that of control sera from unimmunized mice. The statistical significance between pCI/S-300 and S-300 vaccinated groups was determined using Student’s unpaired *t*-test. p-values smaller than 0.05 were considered as statistically significant and indicated with asterisks in the graphs (p<0.01**). Data are shown as geometric mean ± geometric SD.

To further curtail the antigenicity of the NH_2_-terminal domain of the S protein and to determine possible interactions with cellular chaperones, we generated vectors expressing secreted forms of NH_2_-terminal S fragments, namely, pCI/S-300, pCI/S-450, and pCI/S-596, of which only the S-596 antigen covers the entire S-RBD (position S_319–541_) (**Figure 4A**). All three protein fragments were secreted in transiently transfected HEK-293T cells (**Figure 4B**), and we could gain approximately 100 µg of S-300, 20 µg of S-450, and 80–120 µg of S-596 proteins from the cell culture supernatants of 5×10^8^ cells. Only the cell-associated forms of S-300, S-450, and S-596 proteins bound Grp78, but only S-450 and S-596 proteins bound detectable levels of Hsp73 (**Figure 4C**). This was expected, as Hsp73 generally showed a higher binding affinity to larger proteins (53). Furthermore, immunization of mice with pCI/S-596, but not pCI/S-300 or pCI/S-450, induced S-RBD-specific antibodies (**Figure 4D**) and a pronounced pseudovirus neutralization activity (**Figure 4E**). Notably, also the S-300 protein failed to induce detectable levels of S-RBD-specific antibodies and also a neutralization activity (**Figures 4D, E**). However, both the pCI/S-300 and recombinant S-300 induced S-specific serum IgG antibodies (**Figure 4F**). We thus concluded that antibodies directed against the NH_2_-terminal 300 residues of the S protein did not exert a neutralizing activity detectable in our sensitive pseudovirus neutralization assay, supporting the concept that the entire S-RBD was crucial for the induction of neutralizing antibodies.

### 3.3 Priming of S-RBD-specific serum antibody responses in old mice

To determine S-specific immune responses in aging mice, we initially tested the recombinant S6-P_ΔTM_ protein vaccine in young (2–3 months), middle-aged (16 months), and old (23–24 months) mice. Both S-RBD- and S-specific serum antibody titers, and pseudovirus neutralization activities, were similar in young and middle-aged mice, but significantly decreased in old mice (**Supplementary Figure S3**). Therefore, we used 23–24 months old mice to evaluate the age-dependent efficacy of S-specific vaccines.

**Figure 5:**
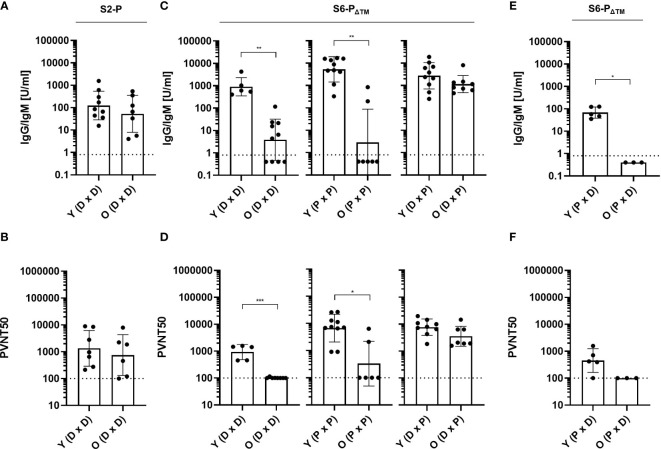
**(A, B)** Young (Y; 2–3 months) and old (O; 23–24 months) C57BL/6J mice were immunized twice (at day 0 and 22) with 100 µg of pCI/S2-P (n=8-9; DxD). **(C, D)** Young (2–3 months) and old (23–24 months) C57BL/6J mice were immunized twice (at day 0 and 22) with 100 µg of pCI/S6-P_ΔTM_ (n=5-10, DxD), 10 µg recombinant S6-P_ΔTM_/Quil-A (n=7-10; PxP), or the heterologous pCI/S6-P_ΔTM_ DNA-prime/S6-P_ΔTM_/Quil-A-protein boost regimen (n=9–10; DxP). **(E, F)** Young (2–3 months) and old (23–24 months) C57BL/6J mice were immunized with 10 µg recombinant S6-P_ΔTM_/Quil-A, followed at day 22 with 100 µg pCI/S6-P_ΔTM_ (n=3–5, PxD). **(A–F)** S-RBD-specific serum antibody titers were determined with a commercial Elecsys Anti-SARS-CoV-2 immunoassay at day 14 after the second vaccination. IgG/IgM antibody titers are shown in U/ml, and the dotted lines represent the limit of quantification (0.8 U/ml) **(A, C, E).** The indicated serum samples were further tested in a vesicular stomatitis virus (VSV)-based SARS-CoV-2 S-carrying pseudovirus assay to determine the neutralizing activity of elicited antibodies. The values are depicted as serum dilution factors that result in 50% pseudovirus neutralization (PVNT50), and the dotted lines represent the detection limit of PVNT50 values (100) **(B, D, F)**. Statistical significance between individual samples between vaccinated young and old mice was determined using Student’s unpaired *t*-test, respectively. p-values smaller than 0.05 were considered as statistically significant and indicated with asterisks in the graphs (p<0.05*, p<0.01**, and p<0.001***). Non-significant differences in groups are not indicated. Data are shown as geometric mean ± geometric SD.

To determine the antigenicity of the monomeric (S6-P_ΔTM_) versus the trimeric S protein (S2-P), we immunized young (2–3 months) and old (23–24 months) mice with pCI/S2-P or pCI/S6-P_ΔTM_ DNAs. Interestingly, the pCI/S2-P vaccine efficiently induced very similar S-RBD-specific antibody titers and pseudovirus neutralization activity in both young and old mice (**Figures 5A, B**). In clear contrast, both the pCI/S6-P_ΔTM_ and the recombinant S6-P_ΔTM_ protein vaccines induced significantly lower S-RBD-specific antibody titers in old than in young mice, along with an overall weakened or absent pseudovirus neutralization activity in the old (**Figures 5C, D**). This highlighted an inefficient priming of neutralizing antibodies in old mice by monomeric S6-P_ΔTM_ antigen.

To determine whether heterologous prime/boost injections of the S6-P_ΔTM_ antigen could enhance its immunogenicity in old mice, we injected groups of young (2–3 months) and old (23–24 months) mice with pCI/S6-P_ΔTM_ DNA followed by a booster injection with recombinant S6-P_ΔTM_ protein (DxP). In young mice, the heterologous DxP vaccination regimen induced very similar S-RBD-specific neutralizing antibody titers as compared to homologous protein-based vaccination (**Figures 5C, D**). Most interestingly, very high S-RBD-specific antibody titers were induced in old mice by heterologous DxP vaccination, which even reached similar levels as in young mice and also conferred a very similar pseudovirus neutralization activity (**Figures 5C, D**). This suggested that heterologous DxP vaccination of old mice primarily affects the quantity but not the quality of the induced antibodies. In contrast, the reversed S6-P_ΔTM_ protein-prime/pCI/S6-P_ΔTM_ DNA-boost (PxD) vaccination could not rescue the impaired antibody response in old mice and also the S-RBD-specific antibody response in young mice was low (**Figures 5E, F**).

We next asked whether the high S-RBD-specific serum antibody titers developed in young and old mice upon S6-P_ΔTM_-specific heterologous DxP vaccination could also cross-protect target cells from pseudoviruses expressing S proteins from different variants of concern (VOCs), namely, Alpha/B.1.1.7, Beta/B.1.351, Delta/B.1.617.2, or Omicron/BA.1 (40, 41, 56). Indeed, S-specific antibodies developed in young and old mice showed a broad cross-neutralization of the different pseudovirus variants, with a weakened recognition and neutralization of Omicron/BA.1 (**Supplementary Figure S4**) (44).

Our findings thus showed that particularly the antigenicity of monomeric S6-P_ΔTM_ antigen was significantly improved in old mice by the heterologous DxP vaccination regimen.

## 4 Discussion

In the COVID-19 pandemic, a multitude of SARS-CoV-2-specific S proteins have been tested for priming protective antibody responses in preclinical animal models and clinical trials. These attempts not only have led to the rapid license and broad usage of RNA- (BNT162b2 and mRNA1273), vector- (e.g., ChAdOx1-S), and protein-based (Novavax) vaccines (57–59) but have also shown a temporal delay for the development of DNA-based vaccines (60). SARS-CoV-2 vaccines largely contain the full-length prefusion-stabilized S protein (S-2P) (47, 48) with a preserved trimeric S-RBD structure (23, 61) and were delivered with optimized nanoparticle and/or adjuvant formulations to improve their stability, cellular uptake, and/or immune-stimulatory activity. As a consequence, vaccines elicited a broad spectrum of T- and B-cell responses and protected recipients from a severe SARS-CoV-2 etiopathology, although the efficacy to induce and/or maintain long-lasting S-specific antibody responses was reduced in old people (8–12, 14, 15, 62–68). This phenomenon of a reduced responsiveness to vaccination in the old population, primarily indicated by weaker priming of antibody titers and their faster decline, was also observed with other, e.g., influenza, vaccines (1, 16, 17). Although strategies to improve vaccine efficacy in old people are limited to simple approaches like increasing the antigen concentration or injection frequencies, they are helpful to enhance and maintain protective antibody responses and decline the age-related immune response heterogeneity to SARS-CoV-2 vaccines (9, 10, 12, 64–67). We here showed that trimeric S2-P elicited high titers of S-RBD-specific antibodies and pseudovirus neutralizing activity in both young and old mice through DNA-based vaccination. In contrast, the S-RBD-specific antibody response was efficiently induced in young but strongly impaired in old mice by the monomeric S6-P_ΔTM_ antigen irrespective from DNA- or protein-based vaccine delivery. The old but not the young immune system thus differentially respond to trimeric and monomeric S proteins. Most interestingly, a simple change in the S6-P_ΔTM_-specific vaccine delivery regimen, i.e., from homologous DNA- or protein-based to heterologous DNA/protein (DxP) vaccination, was sufficient to unleash the reactivity of the old immune system against the monomeric S6-P_ΔTM_ and to induce high titers of S-RBD-specific antibodies and pseudovirus neutralizing activity.

The heterologous DNA-prime/protein-boost (DxP) vaccination approach (69) has been used in preclinical and clinical studies, for example, in the field of HIV, to induce long-lasting, high-affinity antibodies (70–74). Similarly, the DNA-prime/protein-boost strategy was used to generate high affinity monoclonal antibodies for therapy of SARS-CoV-2 variant infection (75). We here showed that the DNA prime was the crucial setting in the S6-P_ΔTM_-specific heterologous DxP vaccination regimen to reconstitute high S-RBD-specific antibody titers in old mice because the reversed PxD vaccination regimen did not. The initial DNA-based antigen expression and antigen presentation in the host might proceed in distinct cell types followed by a specific spatial and temporal sequence of events, which apparently established a beneficial environment for priming T-cell dependent S-RBD-specific antibodies. In an HIV-Env-specific DNA-prime/protein-boost regimen, it was shown that DNA prime was more efficient to induce CD4^+^ T follicular helper cell responses, germinal center (GC) B-cell development, and antigen-specific B-cell responses for both antibody secreting cells and memory B cells (74, 76). This goes along with a more diverse epitope profile, a higher antibody avidity, and an improved neutralizing activity than immunization with only protein or only DNA (72). Particularly in old mice, the specific co-induction of adaptive and innate immune responses, e.g., via toll-like receptor 9 stimulation through CpG motives in the vector DNA, might be crucial for the development of CD4 T cell help, antigen-specific B cells, antibody-producing plasma cells, and/or antigen-specific memory B cells (6). The subsequent exogenous protein booster injection, which unleashes high amounts of antigen in the small injection area, might simultaneously restimulate antigen-experienced T and B cells and thereby induce high antibody titers. However, it remains unknown why the heterologous DxP vaccination regimen strongly stimulates the induction of S-RBD-specific antibodies in old but not in young mice. Qualitative and quantitative B-cell defects apparently played an ancillary role to induce S-RBD-specific antibodies in old mice by the trimeric S2-P, but not the monomeric S6-P_ΔTM_ antigen. We thus speculate that the heterologous S6-P_ΔTM_-specific DxP vaccine might affect distinct B-cell-specific pathways, like the reconstitution of the decreased affinity maturation in old mice (77) and/or specific CD4^+^ T-cell helper functions to reconstitute the humoral immune response in old mice (78). Our studies thus suggest that vaccine-induced B-cell responses benefit from the heterologous S6-P_ΔTM_-specific DNA-prime/protein-boost delivery regimen in old mice, but the underlying molecular mechanism(s) is not yet clarified.

As previously shown for young adolescents, vaccination with S-expressing RNA followed by a subsequent booster with the recombinant Novavax vaccine (59) led to fewer breakthrough infections and generated higher antibody and T-cell responses against wild-type and omicron variants than homologous mRNA vaccination (79). This implied that a heterologous mRNA/protein boost regimen is more efficient to induce humoral immune responses than the homologous mRNA/mRNA vaccine. Furthermore, several studies showed not only an enhanced and more sustained SARS-CoV-2 S- but also influenza hemagglutinin (HA)-specific antibody response in old individuals induced through heterologous mRNA-prime/protein-boost regimens as compared to homologous mRNA vaccination (80–83). Interestingly, there was also increasing evidence that different antigen-specific heterologous prime/boost strategies might also improve the efficacy of currently available vaccines. Initial findings came from adenoviral vector (ChAdOx1-nCov-19) vaccinated individuals, who, after reports of thromboembolic events, received second or third booster injections with heterologous mRNA-based vaccines. The major outcome of these studies was that both mRNA and ChAdOx1-nCov-19 vaccines boosted the initial ChAdOx1-nCov-19-induced immunity, but the heterologous mRNA booster was more effective to enhance and sustain cellular T- and humoral B-cell responses against wild-type SARS-CoV-2 and different VOCs (40, 41, 84–88). However, the underlying molecular mechanisms are also unknown.

Using Strep-tag purification of cell-associated S proteins, we could show that *de novo* expressed S proteins, but not unrelated antigens (42), stably bound to Grp78 and Hsp73. In line with this, it has been shown that the SARS-CoV-2 S protein could interact with Grp78 and form a trimeric complex with ACE2, indicating that SARS-CoV-2 virions utilize Grp78 as an auxiliary host factor for viral entry and infection (89, 90). We previously showed that the stable binding of an antigen to cellular Hsp73 significantly enhanced the humoral and cellular immune response upon DNA vaccination, confirming the role of the Hsp73 chaperone as endogenous adjuvants (51–53). In particular, the stable binding of S proteins to cellular Hsp73 and/or Grp78 might affect the magnitude of humoral and cellular S-specific immune responses by nucleic-acid-based vaccines. Such an endogenous adjuvant function might explain the efficient priming of antibody responses in young and old mice by DNA-based vaccines that express Hsp73- and Grp78-bound S and S2-P proteins.

Overall, our findings show a clear advantage of using a trimeric vs. a monomeric S antigen to induce high S-RBD-specific antibody titer and functional neutralization activity in old mice. The age-associated impairment to prime S-RBD-specific antibodies with the monomeric S6-P_ΔTM_ antigen upon homologous DNA and protein vaccination can be reconstituted by delivering the vaccine in a heterologous (DxP) immunization regimen. This clearly showed that both the antigen structure and the delivery platform are crucial to efficiently prime humoral immune responses in old mice and might be relevant for designing age-adapted vaccine strategies.

## Data availability statement

The original contributions presented in the study are included in the article/**Supplementary Material**. Further inquiries can be directed to the corresponding author.

## Ethics statement

The animal study was performed in accordance to the National Animal Welfare Law and reviewed/approved by the Committee on the Ethics of Animal Experiments of the University of Ulm (Tierforschungszentrum der Universität Ulm) and the Regierungspräsidium Tübingen (REFERAT 35-Veterinärwesen, Lebensmittelüberwachung). The study was conducted in accordance with the local legislation and institutional requirements.

## Author contributions

DP, AS, FK, RG, LK, JK, and KS performed experiments and researched and interpreted data. SK, JM, and RS conceived the experiments, secured funding, and discussed and interpreted data. DP, KS, and RS wrote the manuscript. All authors edited and approved the final version of the manuscript.
