# Supplementary material for: Heterologous DNA-prime/protein-boost immunization with a monomeric SARS-CoV-2 spike antigen redundantizes the trimeric receptor-binding domain structure to induce neutralizing antibodies in old mice
Source: Front Immunol. 2023 Sep 11;14:1231274. doi: 10.3389/fimmu.2023.1231274 (PMC10518615; doi:10.3389/fimmu.2023.1231274)
Supplement: Supplementary file 1 [file DataSheet_1.pdf]

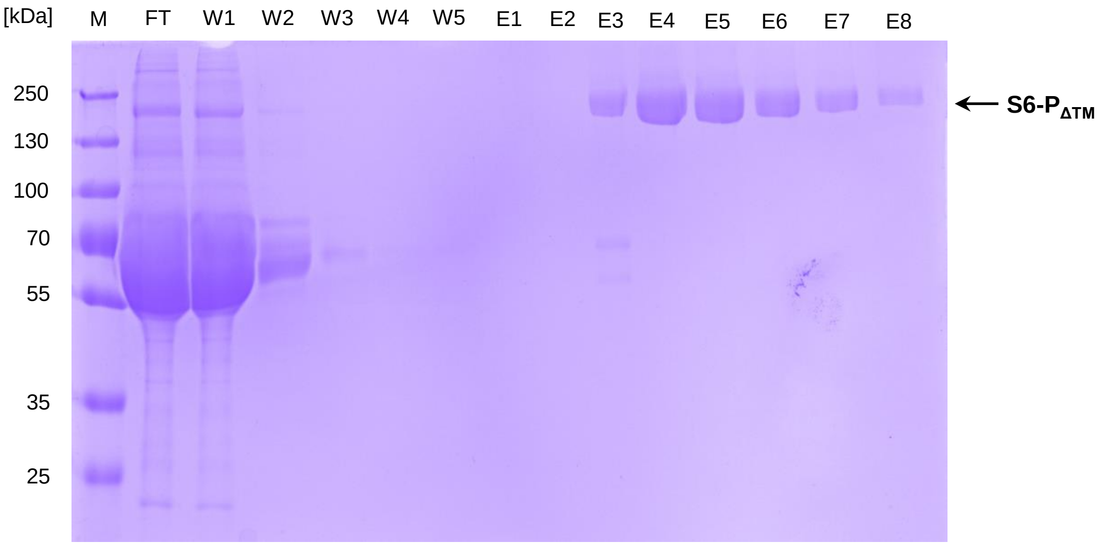

Supplementary Figure S1.

Purification of the recombinant S6-P$_{\Delta TM}$.
HEK-293T cells were transiently transfected with the pCI/S6-P$_{\Delta TM}$ vector and cell culture supernatants were used for Strep-Tactin-specific purification. Samples from each purification step (flow through, FT; washing steps, W1-W5) and elution fractions (E1-E8) were subjected to SDS-PAGE followed by Coomassie Brillant Blue staining of the gels. The position of the S6-P$_{\Delta TM}$ protein is indicated with an arrow.

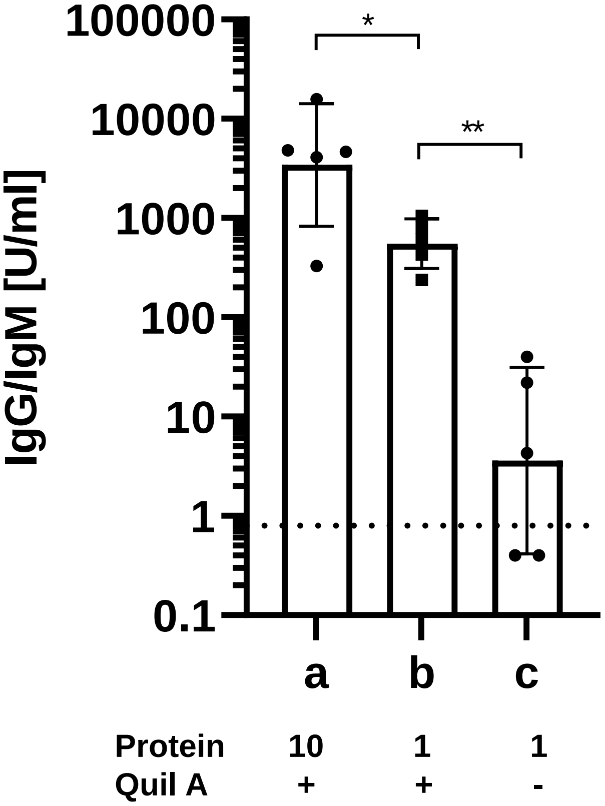

Supplementary Figure S2.

Testing recombinant S6-P$_{\Delta TM}$ protein immunization in young mice
Young (2-3 months) C57BL/6J mice were immunized twice (at day 0 and 22) with either 10 µg S6-P$_{\Delta TM}$/Quil-A (group a), 1 µg S6-P$_{\Delta TM}$/Quil-A (group b), or 1 µg S6-P$_{\Delta TM}$ (group c) (n=5). S-RBD specific serum antibody titers were determined with a commercial Elecsys anti SARS-CoV-2 S immunoassay at day 14 after the final vaccination. IgG/IgM antibody titers are shown in U/ml. Data are shown as geometric mean $\pm$ geometric SD. To show that the protein amount as well as the adjuvant had significant influence in the induction of RBD-specific antibodies, the statistical significance between 10 and 1 µg S6-P$_{\Delta TM}$/Quil-A vaccines (group a and b, effect of dose), and between 1 µg S6-P$_{\Delta TM}$ Quil-A^{+} and Quil-A^{-} vaccines (group b and c, effect of adjuvant), was determined using Student's unpaired *t*-test. P-values smaller than 0.05 were considered as statistically significant and indicated with asterisks in the graphs ($p<0.05$*, $p<0.01$**). Dotted lines represent the limit of quantification (0.8 U/ml).

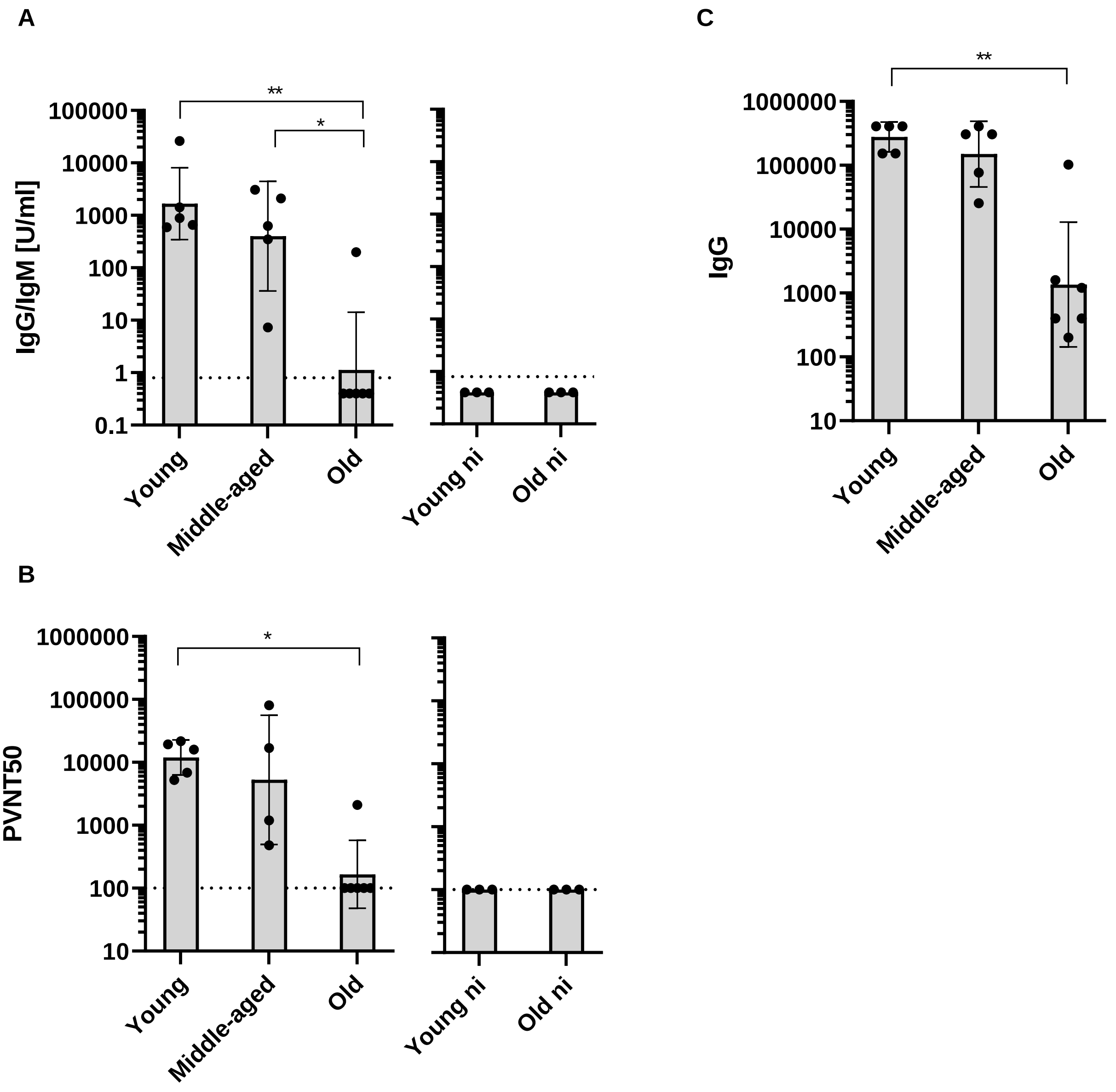

Supplementary Figure S3.

Testing recombinant S6-P$_{\Delta TM}$ protein immunization in aging mice.
Young (2-3 months), middle-aged (16 months) and old (23-24 months) C57BL/6J mice (n=5) were immunized twice (at day 0 and 22) with 10 µg S6-P$_{\Delta TM}$/Quil-A. As a control, we used three blood samples from unimmunized young (Young ni) and old (Old ni) mice. **(A)** S-RBD-specific serum antibody titers were determined with a commercial Elecsys anti-SARS-CoV-2 S immunoassay at day 14 after the second vaccination. IgG/IgM antibody titers are shown in U/ml. Dotted lines represent the limit of quantification (0.8 U/ml). **(B)** Serum samples were further tested in a vesicular stomatitis virus (VSV)-based SARS-CoV-2 S-carrying pseudovirus system to determine the neutralizing activity of elicited antibodies. The values are depicted as serum dilution factors that result in 50% pseudovirus neutralization (PVNT50). Dotted lines represent the limit of detection (100). **(C)** Furthermore, S-specific IgG serum antibody titers were determined by end point ELISA using a recombinant S6-P$_{\Delta TM/EPEA}$ detection antigen. The S-specific endpoint titers shown are defined as the highest serum dilution that resulted in an absorbance value three times greater than that of control sera from non-immunized mice. **(A-C)** The statistical significance between all groups was determined using Kruskal-Wallis test followed by Dunn's multiple comparisons test. P-values smaller than 0.05 were considered as statistically significant. Only statistically significant differences were indicated with asterisks in the graphs ($p<0.05$* and $p<0.01$**). Data are shown as geometric mean ± geometric SD.

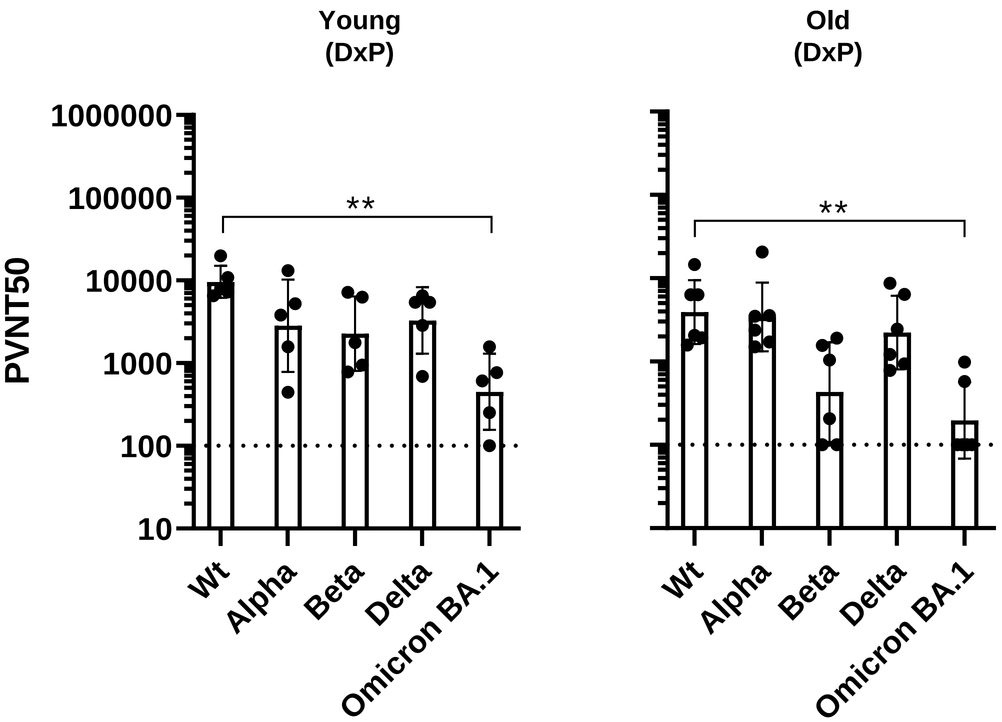

Supplementary Figure S4.

Cross-neutralization of different VOCs by S-specific antibodies.
Young (2-3 months) and old (23-24 months) C57BL/6J mice (n=5-6) were primed with pCI/S6-$P_{\Delta TM}$ DNA and boosted with S6-$P_{\Delta TM}$/Quil-A protein (DxP) at day 22. Sera from immunized mice were collected 14 days after the second immunization and analyzed in a vesicular stomatitis virus (VSV)-based SARS-CoV-2 pseudovirus assay, carrying different S-proteins from wild-type (Wuhan-Hu-1 strain, wt) or different variants of concern (VOCs), namely Alpha/B.1.1.7, Beta/B.1.351, Delta/B.1.617.2 or Omicron/BA.1. The values are depicted as serum dilution factors that result in 50% pseudovirus neutralization (PVNT50). The statistical significance between the groups was determined using Kruskal-Wallis test followed by Dunn's multiple comparisons test. P-values smaller than 0.05 were considered as statistically significant. Only statistically significant differences were indicated with asterisks in the graphs ($p<0.01$**). Dotted lines represent the limit of detection (100).
